# Reversal of Endogenous Bioelectrical Network Collapse in Advanced Childhood Cerebral X-Linked Adrenoleukodystrophy

**DOI:** 10.3390/neurolint18040063

**Published:** 2026-03-24

**Authors:** Salvatore Rinaldi, Arianna Rinaldi, Vania Fontani

**Affiliations:** 1 Department of Reparative and Regenerative Medicine, Rinaldi Fontani Institute, 50144 Florence, Italy; ari@irf.it (A.R.); vfontani@irf.it (V.F.); 2 Department of Adaptive Neuro Psycho Physio Pathology and Neuro Psycho Physical Optimization, Rinaldi Fontani Institute, 50144 Florence, Italy; 3 Research Department, Rinaldi Fontani Foundation, 50144 Florence, Italy

**Keywords:** childhood cerebral X-linked adrenoleukodystrophy (cALD), electroencephalography (EEG), quantitative EEG (qEEG), independent component analysis (ICA), sLORETA, endogenous bioelectrical activity, network reorganisation, paediatric neurodegeneration, REAC neuroregenerative treatment, radio electric asymmetric conveyor (REAC)

## Abstract

Background/Objectives: Advanced childhood cerebral X-linked adrenoleukodystrophy (cALD) is traditionally regarded as an irreversible terminal phase of neurodegeneration driven by inflammatory demyelination and axonal loss. Experimental evidence indicates that endogenous bioelectrical fields regulate central nervous system organisation, raising the possibility that functional network collapse in cALD may be biologically modifiable, even in the presence of persistent structural damage. This study examined whether longitudinal modulation of endogenous bioelectrical network organisation is associated with sustained clinical and neurophysiological stabilisation in advanced cALD. Methods: We performed a longitudinal observational analysis of two paediatric patients with advanced childhood cerebral X-linked adrenoleukodystrophy undergoing repeated neuroregenerative treatment cycles. Standardised scalp electroencephalography was recorded during spontaneous wakefulness and repeated over months under comparable vigilance conditions. Multimodal analysis included conventional EEG, quantitative EEG, independent component analysis, and standardised low-resolution electromagnetic tomography (sLORETA). Clinical function was assessed using validated measures of consciousness, swallowing, and voluntary motor behaviour. Results: Across patients, longitudinal recordings demonstrated sustained stabilisation of consciousness, swallowing, and voluntary motor function, accompanied by reproducible reorganisation of pathological brain rhythms. Delta and theta oscillations showed a consistent topographical redistribution from limbic–frontoinsular networks towards sensorimotor and parietal integrative cortices. These changes were observed across modalities and timepoints and are unlikely to reflect spontaneous fluctuation, delayed effects of haematopoietic stem cell transplantation, or state-dependent EEG variation. Conclusions: Advanced childhood cerebral X-linked adrenoleukodystrophy is associated with disorganisation of endogenous bioelectrical network activity. In this longitudinal analysis, large-scale network reorganisation was temporally associated with sustained clinical stabilisation, supporting a view of late-stage cALD as a dynamic disorder of network-level vulnerability, rather than a fixed terminal state.

## 1. Introduction

Childhood cerebral X-linked adrenoleukodystrophy (cALD) is one of the most aggressive paediatric neurodegenerative disorders, characterised by inflammatory demyelination, rapid network disintegration, and early loss of consciousness, motor control, and environmental interaction. Once cerebral involvement becomes clinically manifested, prognosis is poor even after haematopoietic stem cell transplantation (HSCT), and many patients continue to deteriorate despite suppression of the primary metabolic and immunological drivers of the disease [1,2,3]. This dissociation between structural pathology and functional outcome indicates that additional layers of neurobiological regulation critically shape disease progression.

While magnetic resonance imaging captures the anatomical extent of demyelination, it does not account for the profound collapse of functional integration that defines advanced cALD. In contrast, electroencephalography (EEG) consistently reveals severe disruption of cortico–subcortical coordination, including diffuse slowing, loss of physiological rhythms, epileptiform activity, and breakdown of background organisation [4,5]. These electrophysiological abnormalities closely parallel loss of arousal, voluntary behaviour, and consciousness [6], and they typically worsen in parallel with clinical decline. Importantly, spontaneous recovery of organised EEG activity has not been described in advanced cALD, even when structural progression slows.

To date, available EEG reports in childhood cerebral ALD have primarily described single-timepoint electrophysiological abnormalities, most often in relation to seizure activity or acute clinical states, whereas longitudinal characterisation of evolving EEG or quantitative EEG network organisation over time has not been reported [4,5].

A growing body of work in developmental neurobiology and regenerative neuroscience has established that endogenous bioelectrical fields act as master regulators of central nervous system organisation, integrating molecular signalling, inflammation, and large-scale network coordination [7,8]. These bioelectrical gradients are not epiphenomena of neuronal firing but constitute an organising layer that integrates molecular, cellular, and network-level processes, the disruption of which leads to progressive loss of hierarchical control, limbic–autonomic dominance, and encephalopathic states that may evolve partially independently of structural injury.

Within this framework, advanced childhood leukodystrophy can be conceptualised not as a biologically fixed degenerative endpoint, but as a state of endogenous bioelectrical network collapse. If this hypothesis is correct, restoration of coherent bioelectrical patterning should permit partial reintegration of residual neural networks, even in the presence of ongoing white matter pathology.

Radio electric asymmetric conveyor (REAC) neuroregenerative treatment (RGN-N) is a standardised, non-invasive intervention designed to interact with altered endogenous bioelectrical activity through asymmetrically conveyed radioelectric fields. Rather than imposing external electrical currents, this approach aims to modulate the spatial organisation of pathological endogenous bioelectrical patterns. Preclinical studies have shown that modulation of endogenous bioelectric fields can influence neuroinflammation, cellular metabolism, and functional recovery in models of neurodegeneration, and recent paediatric observations have reported preliminary stabilisation and functional gains in severe leukodystrophies [9].

**Figure 1:**
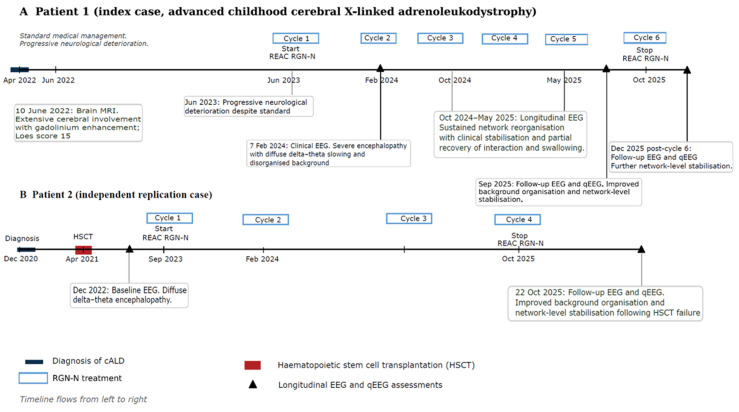
Longitudinal clinical timelines and neurophysiological assessments in advanced childhood cerebral X-linked adrenoleukodystrophy. (**A**) Index patient, showing diagnosis, standard medical management, and progressive neurological deterioration prior to the initiation of repeated REAC neuroregenerative (RGN-N) treatment cycles. Serial longitudinal EEG and quantitative EEG (qEEG) assessments demonstrate a durable large-scale reorganisation of endogenous brain rhythms temporally associated with sustained clinical stabilisation. (**B**) Independent replication case with prior haematopoietic stem cell transplantation (HSCT) performed without clinical or neurophysiological benefit, followed by continued neurological deterioration. Subsequent initiation of REAC RGN-N treatment cycles is associated with longitudinal EEG evidence of background reorganisation and network-level stabilisation. Timelines are aligned chronologically from left to right. EEG and qEEG recordings were acquired without sedatives and under stable clinical and pharmacological conditions. In the index patient, detailed quantitative EEG, ICA, and sLORETA analyses compare recordings obtained prior to the sixth RGN-N cycle (October 2025) and approximately two months after the sixth cycle (December 2025).

Here, we report a longitudinal analysis of two independent paediatric patients with advanced cALD treated with repeated REAC RGN-N cycles, integrating clinical outcomes with conventional EEG, quantitative EEG, independent component analysis (ICA), and standardised low-resolution electromagnetic tomography (sLORETA). We demonstrate that, in contrast to the expected natural history of relentless deterioration, these patients exhibit sustained clinical stabilisation accompanied by reproducible reorganisation of endogenous brain rhythms. Most notably, we identify a consistent topographical redistribution of slow-wave activity from limbic–degenerative networks towards sensorimotor–integrative cortical systems, providing converging clinical and neurophysiological evidence that endogenous bioelectrical network collapse in paediatric neurodegeneration is not only detectable but potentially biologically modifiable. To place these observations within a clear temporal and clinical framework, the longitudinal clinical course, therapeutic exposures, and neurophysiological assessments of the patients are summarised in Figure 1.

## 2. Materials and Methods

### 2.1. Patients and Study Design

This study reports a longitudinal observational analysis of two independent paediatric cases with advanced childhood cerebral X-linked adrenoleukodystrophy (cALD) treated with repeated cycles of REAC neuroregenerative (RGN-N) therapy in the context of progressive neurological deterioration despite standard disease management. Both patients had genetically confirmed X-linked adrenoleukodystrophy with established cerebral involvement and severe neurological disability at the time of initiation of REAC treatment.

At the time of initiation of REAC RGN-N treatment, both patients exhibited advanced neurological impairment, including severe loss of voluntary motor control, impaired consciousness or environmental interaction, dysphagia, spasticity, and electroencephalographic evidence of diffuse encephalopathy. In one patient, haematopoietic stem cell transplantation (HSCT) had been performed without demonstrable clinical or neurophysiological benefit; in the other patient, no HSCT was undertaken. In both cases, continued neurological deterioration was documented prior to the initiation of REAC RGN-N treatment.

The intervention consisted of standardised cycles of REAC neuroregenerative treatment (RGN-N) delivered using the REAC BENE mod 110 medical device (ASMED, Scandicci, Italy), configured exclusively for the RGN-N protocol. Each treatment cycle was administered over approximately 48 h, typically delivered as 6–8 h of continuous treatment per day, using asymmetric conveyor probes positioned along the spinal axis according to the protocol. All stimulation parameters were pre-set and could not be modified by the operator, ensuring reproducibility and consistency across patients and treatment cycles.

Longitudinal clinical follow-up was conducted by multidisciplinary teams including neurologists, physiatrists, neurophysiologists, and therapists, together with continuous caregiver observation. Clinical outcomes were assessed using standardised scales appropriate to disease severity, including the Functional Oral Intake Scale (FOIS), Trunk Control Measurement Scale (TCMS), Modified Ashworth Scale (MAS), and the Coma Recovery Scale—Revised (CRS-R), as well as structured assessment of consciousness and environmental interaction.

Neurophysiological evaluation included conventional electroencephalography (EEG) and quantitative EEG (qEEG) in both patients. Independent component analysis (ICA) and standardised low-resolution electromagnetic tomography (sLORETA) were available in one patient, allowing detailed longitudinal assessment of network-level organisation. Recordings were obtained at predefined longitudinal time points before and after specific RGN-N cycles, enabling evaluation of spectral, topographical, and network-level changes over time.

All EEG recordings were acquired in spontaneous wakefulness, without sedatives, recent changes in antiepileptic or psychoactive medications, or sleep-deprivation protocols, and were repeated under comparable vigilance conditions. EEG data were analysed by experienced neurophysiologists using standardised clinical and quantitative criteria, independent of treatment delivery. The persistence of neurophysiological changes across recordings separated by months allowed assessment of the durability and directionality of network reorganisation, excluding transient or state-dependent effects.

### 2.2. Neurophysiological Recording and Analysis

Standardised scalp electroencephalography (EEG) was performed in both patients. Recordings were obtained in spontaneous wakefulness, without sedatives, recent changes in antiepileptic or psychoactive medications, or sleep-deprivation protocols, and were repeated under comparable vigilance conditions. EEG was acquired at predefined longitudinal time points and during follow-up using age-appropriate montages and standard international electrode placement, with systematic minimisation of movement, muscle, and environmental artifacts.

EEG data were analysed by experienced neurophysiologists using standardised clinical and quantitative criteria, independent of treatment delivery and without knowledge of the study hypotheses. The persistence of electrophysiological changes across recordings separated by months allowed exclusion of transient arousal- or state-dependent effects.

Quantitative EEG (qEEG) analysis was performed using validated brain-mapping software (Mitsar-201 system and winEEG software (Mitsar Co. Ltd., St. Petersburg, Russia; official website: https://mitsar-eeg.com/eeg-system-solutions/wineeg-research-software/; accessed on 9 March 2026), including LORETA/sLORETA source localization modules (The KEY Institute for Brain-Mind Research, University of Zurich, Zurich, Switzerland; official website: https://www.uzh.ch/keyinst/NewLORETA/sLORETA/sLORETA.htm; accessed on 9 March 2026)) to assess spectral power distribution, hemispheric symmetry, and regional frequency composition. In one patient, independent component analysis (ICA) was applied to decompose EEG signals into spatially independent generators, and standardised low-resolution electromagnetic tomography (sLORETA) was used to estimate the cortical localisation of dominant oscillatory sources.

This integrated EEG–qEEG–ICA–sLORETA framework enabled both global and regional assessment of brain network organisation, with particular attention to delta and theta band activity, posterior dominant rhythm expression, hemispheric organisation, and the spatial redistribution of pathological slow-wave generators over time. Recordings obtained before and after specific RGN-N cycles were separated by months rather than days, allowing evaluation of the durability and directionality of network-level reorganisation rather than transient or state-dependent fluctuations.

Earlier clinical and neurophysiological assessments of the index patient, including baseline and intermediate treatment phases, have been previously reported in a single-patient case report [10]. In that study, EEG and quantitative EEG findings were documented across initial and intermediate treatment cycles. The present manuscript deliberately focusses on a later longitudinal phase of the same patient, analysing changes observed between the fifth and sixth REAC RGN-N treatment cycles, when the disease had already reached an advanced and functionally irreversible stage. Accordingly, the functional and neurophysiological status at the fifth treatment cycle constitutes the reference point for longitudinal comparison in the present study.

### 2.3. Clinical Assessment

Clinical status was assessed longitudinally using standardised neurological and functional scales appropriate to disease severity. Swallowing function was evaluated using the Functional Oral Intake Scale (FOIS), postural control with the Trunk Control Measurement Scale (TCMS), spasticity with the Modified Ashworth Scale (MAS), and level of consciousness and recovery from disorders of consciousness with the Coma Recovery Scale—Revised (CRS-R). Assessments were performed by clinicians experienced in paediatric neurorehabilitation and severe brain injury. Clinical changes were documented longitudinally and corroborated by independent reports from caregivers and therapists involved in daily care, allowing confirmation of the persistence and functional relevance of observed changes.

## 3. Results

### 3.1. Clinical Evolution

Across both paediatric patients with advanced childhood cerebral X-linked adrenoleukodystrophy, longitudinal follow-up was associated with sustained clinical stabilisation and, in selected domains, partial recovery of neurological functions typically lost at this stage of disease.

Prior to the initiation of REAC neuroregenerative (RGN-N) treatment, both patients exhibited progressive neurological deterioration despite standard supportive care; in one patient, neurological decline continued following haematopoietic stem cell transplantation (HSCT) performed without clinical benefit. Baseline clinical status was characterised by severe impairment of consciousness, swallowing, voluntary motor control, and environmental interaction.

Following repeated RGN-N treatment cycles, neither patient showed the relentless clinical decline that typically characterises advanced cALD. Instead, longitudinal observation documented stabilisation or reproducible improvements in arousal, visual engagement, voluntary motor initiation, and swallowing capacity.

In one patient, formal Coma Recovery Scale—Revised (CRS-R) assessment demonstrated a transition from a persistent vegetative state to emergence from the minimally conscious state, with consistent visual fixation, tracking, emotional responsiveness, and reproducible voluntary motor responses. In the same patient, Functional Oral Intake Scale (FOIS) scores improved from no oral intake (level 1) to partial oral feeding (levels 4–5), and spasticity showed a clinically meaningful reduction on the Modified Ashworth Scale, facilitating daily care and interaction.

In the second patient, clinical evolution was characterised by sustained stabilisation of swallowing function, postural control, and spasticity, without further neurological decline. These clinical changes were maintained over months following each RGN-N cycle.

No new pharmacological, surgical, or intensive rehabilitative interventions were introduced during the observation period.

### 3.2. Electrophysiological Reorganisation

**Figure 2:**
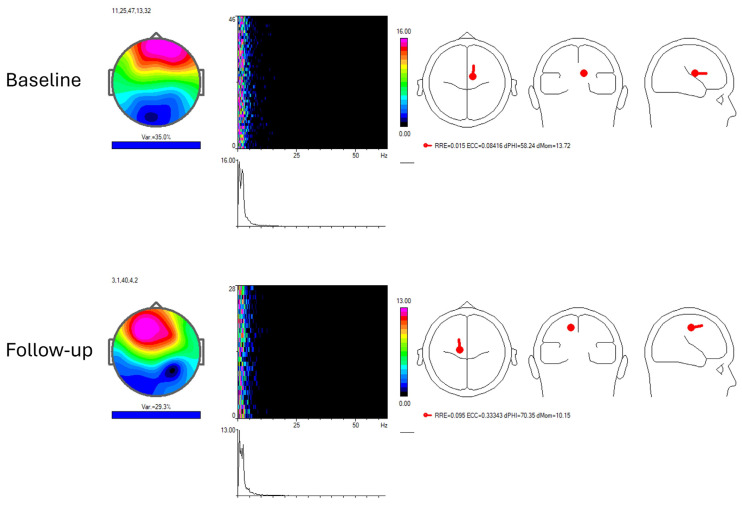
Longitudinal EEG and quantitative EEG component-level analysis in the index patient comparing the initial longitudinal evaluation (October 2025, prior to the sixth RGN-N cycle) and follow-up assessment approximately two months after the sixth cycle (December 2025). Representative independent components derived from EEG recordings at the first longitudinal evaluation and at follow-up are shown. At the first longitudinal EEG, dominant slow-wave activity is associated with disorganised background rhythms and impaired physiological organisation. At follow-up, EEG and qEEG demonstrate re-emergence of structured background activity and redistribution of spectral power towards a more organised cortical pattern, consistent with durable electrophysiological reorganisation rather than transient state-dependent modulation. Recordings shown are separated by several months, excluding transient arousal-related effects.

At the initial longitudinal EEG evaluation (October 2025, prior to the sixth RGN-N cycle), recordings in both patients demonstrated severe encephalopathy characterised by diffuse delta–theta slowing, loss of physiological background rhythms, hemispheric disorganisation, and, in one case, limbic-dominant slow-wave activity, as illustrated in Figure 2.

These findings were consistent with profound cortico–subcortical dysfunction typical of advanced cerebral ALD.

During longitudinal follow-up, both patients exhibited reproducible changes in EEG organisation. Conventional EEG demonstrated re-emergence of posterior dominant rhythms, improved hemispheric symmetry, increased background continuity, and reduction of diffuse slow-wave predominance. Previously documented irritative or epileptiform features disappeared or became markedly attenuated.

Quantitative EEG analysis confirmed these findings, showing redistribution of spectral power towards more physiological frequency bands and restoration of posterior cortical predominance. These electrophysiological changes persisted across recordings separated by months, indicating durable reorganisation rather than transient state-dependent modulation.

### 3.3. Network-Level Reallocation of Slow-Wave Activity

**Figure 3:**
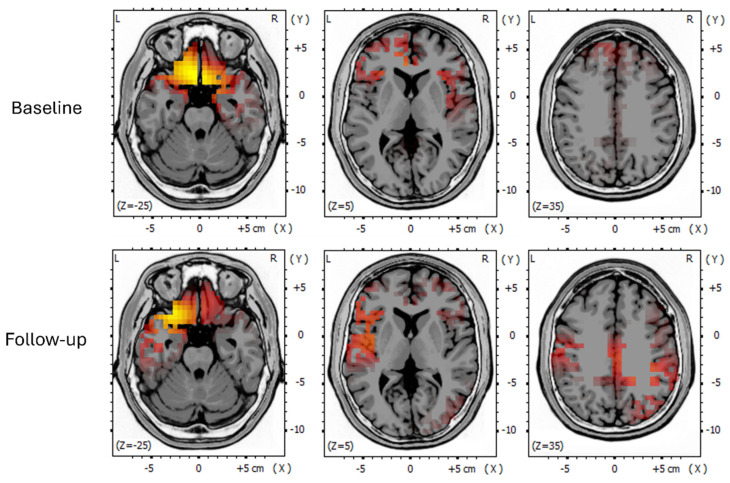
Longitudinal EEG source localisation analysis with MRI coregistration in the index patient. sLORETA maps of EEG current density are overlaid on the individual MRI template at the first longitudinal EEG evaluation (upper row) and at follow-up (lower row). The images show representative axial slices at different stereotactic levels (z = −25, z = 5, and z = 35). L and R indicate the left and right hemispheres, respectively; X, Y, and Z denote stereotactic coordinates. Warmer colours (from red to yellow) indicate higher estimated current density within the displayed source distribution. At the first longitudinal assessment, dominant slow-wave generators are primarily localised to fronto-limbic and paralimbic regions. At follow-up, slow-wave activity is no longer limbic-dominant but is redistributed towards sensorimotor and parietal integrative cortices, indicating a network-level reallocation of pathological oscillatory activity rather than suppression of slow rhythms. This topographical reorganisation parallels sustained clinical stabilisation and improved environmental interaction.

In the index patient, longitudinal EEG source localisation analysis provided direct insight into the spatial organisation of pathological slow-wave activity at the network level, as shown in Figure 3.

At the initial longitudinal qEEG/ICA evaluation (October 2025, prior to the sixth RGN-N cycle), recordings demonstrated dominant delta–theta generators localised predominantly to fronto-limbic and paralimbic regions, including Brodmann areas 11, 25, 47, 13, and 32, consistent with marked limbic network dominance.

At follow-up evaluation approximately two months after the sixth RGN-N cycle (December 2025), the same frequency components remained detectable but exhibited a substantially modified spatial organisation. Source localisation revealed a reduction of fronto-limbic predominance, with redistribution of slow-wave activity towards primary and secondary sensorimotor and parietal integrative cortices, including Brodmann areas 1, 2, 3, 4, and 40.

Importantly, this longitudinal change reflected reorganisation of the spatial distribution of slow-wave generators rather than suppression of low-frequency activity. Overall spectral content remained comparable between the pre-sixth-cycle and post-sixth-cycle assessments, whereas the topographical organisation of oscillatory activity shifted from a limbic-dominant pattern towards a broader cortical distribution.

This network-level reallocation of slow-wave activity occurred in parallel with sustained clinical stabilisation and improved environmental interaction, providing convergent electrophysiological evidence of adaptive bioelectrical network remodelling in advanced paediatric cerebral X-linked adrenoleukodystrophy. These findings describe longitudinal clinical and neurophysiological associations within the limits of an observational design.

### 3.4. Replication Case: Longitudinal EEG Evidence of Convergent Network Reorganisation

**Figure 4:**
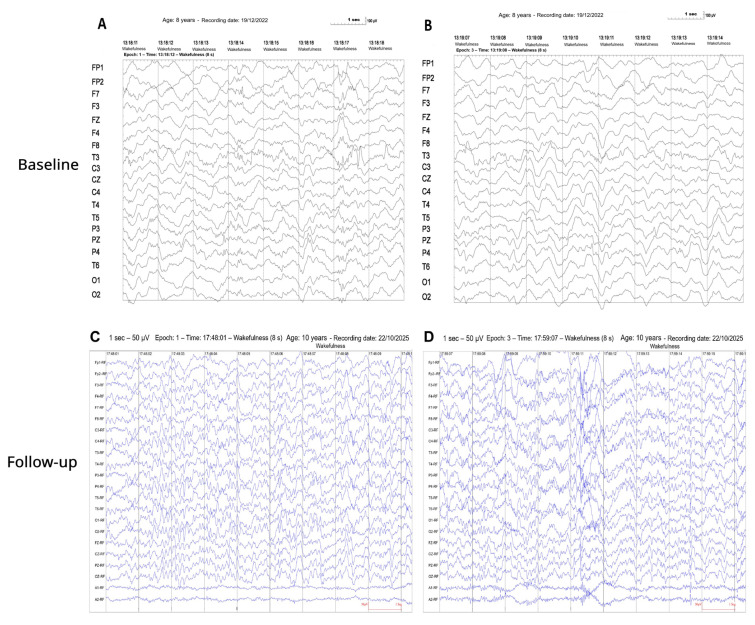
Replication case, baseline-to-follow-up EEG comparison. Baseline electroencephalogram (EEG) recorded on 19 December 2022: (**A**,**B**) representative wakefulness EEG epochs showing diffusely slowed background activity with predominance of delta–theta rhythms (4–6 Hz), marked background disorganisation, and absence of a stable posterior dominant rhythm, consistent with severe cortical dysfunction in advanced childhood cerebral X-linked adrenoleukodystrophy. Follow-up EEG recorded on 22 October 2025 after repeated REAC RGN-N treatment cycles: (**C**,**D**) representative wakefulness EEG epochs showing reduced diffuse slow-wave predominance, improved channel differentiation, and partial re-emergence of more organised background activity, consistent with longitudinal electrophysiological reorganisation.

To evaluate whether the neurophysiological changes observed in the index case reflected an idiosyncratic phenomenon or a reproducible pattern, a second independent patient with advanced childhood cerebral X-linked adrenoleukodystrophy was analysed using the same longitudinal neurophysiological framework. At the time of assessment, the replication patient was at a disease stage comparable to that of the index case, characterised by advanced functional impairment and severe disruption of consciousness and motor function. A baseline electroencephalogram (EEG) performed on 19 December 2022 revealed a severely abnormal tracing characterised by diffuse delta–theta slowing (4–6 Hz), marked disorganisation of background activity, and absence of a physiological posterior dominant rhythm. Interictal irritative activity was present in the left anterior–middle temporal region, indicating cortical instability. These findings were consistent with severe cortico-subcortical dysfunction and advanced cerebral involvement (Figure 4, upper row). During subsequent longitudinal follow-up across successive REAC RGN-N treatment cycles, representative wakefulness EEG segments recorded on 22 October 2025 showed reduction in diffuse slow-wave predominance, improved background differentiation, and partial re-emergence of more organised cortical activity (Figure 4, lower row).

**Figure 5:**
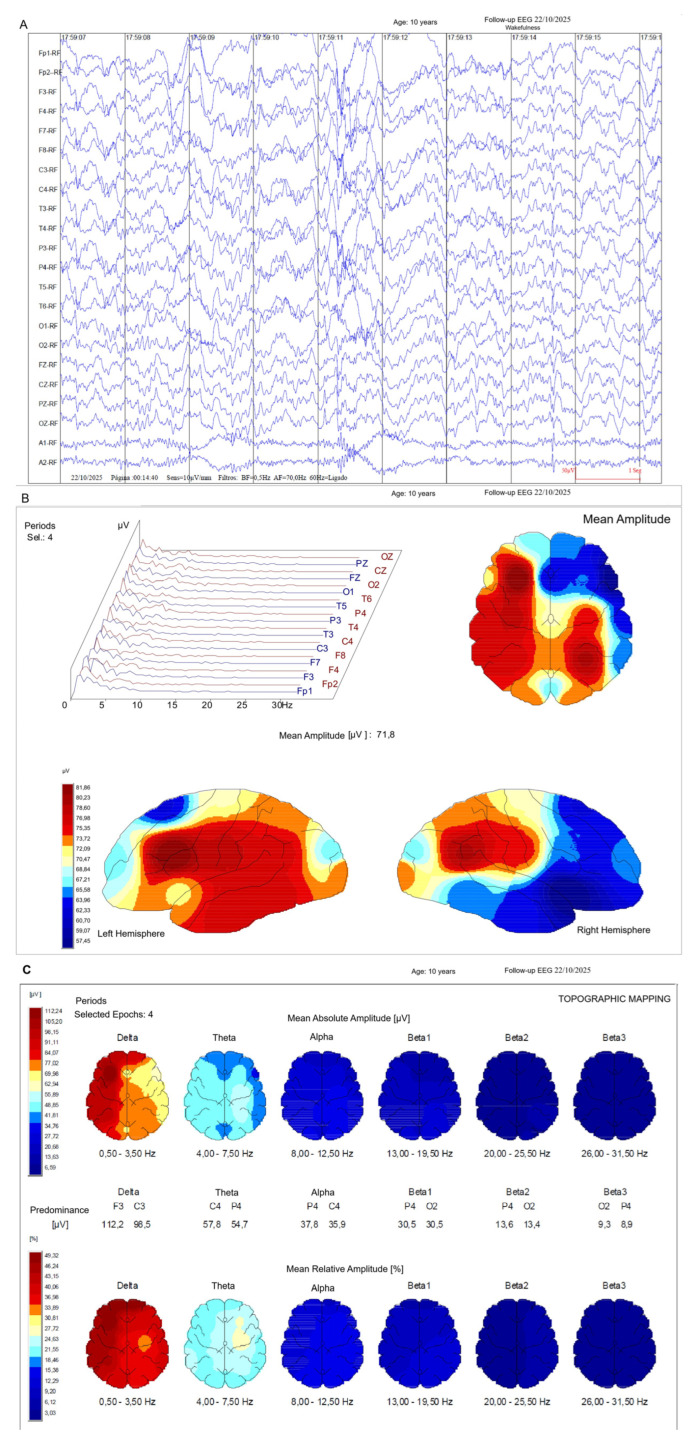
Replication case—longitudinal qEEG and network-level changes. Quantitative EEG analyses were performed after completion of the REAC RGN-N treatment cycles in the replication case. (**A**) Power spectral distribution showing reduced diffuse slow-wave predominance. (**B**) Topographical qEEG maps illustrating improved spatial differentiation of cortical activity. (**C**) Network-level analysis indicating partial restoration of organised functional connectivity patterns.

Quantitative EEG analysis further demonstrated a redistribution of spectral power and improved spatial organisation, mirroring the network-level changes documented in the index case (Figure 5).

The convergence of qualitative EEG and quantitative EEG findings across two independently assessed patients supports the reproducibility of the observed pattern of endogenous brain network reorganisation and argues against a coincidental or patient-specific phenomenon.

## 4. Discussion

This longitudinal multi-case analysis provides converging clinical and neurophysiological evidence that advanced childhood cerebral X-linked adrenoleukodystrophy is associated with a state of endogenous bioelectrical network disorganisation, rather than representing a biologically immutable terminal phase of structural degeneration. Across independent patients, repeated REAC neuroregenerative (RGN-N) treatment was temporally associated with sustained clinical stabilisation and, in selected domains, partial recovery of functions typically lost at this disease stage, together with reproducible reorganisation of pathological brain rhythms. These observations emerge in a clinical context widely regarded as functionally irreversible.

Prior EEG descriptions in childhood cerebral ALD have largely been limited to single-timepoint observations, most often reported in relation to seizure activity or acute clinical states, and have not addressed the longitudinal evolution of EEG or quantitative EEG network organisation over time. The absence of such longitudinal investigations reflects the prevailing assumption that functional improvement at advanced disease stages is biologically unattainable.

Consistent with prior clinical experience, advanced cerebral involvement in childhood ALD remains associated with poor functional prognosis even after haematopoietic stem cell transplantation, with continued neurological deterioration despite suppression of the primary metabolic and immunological drivers of the disease. The inclusion of a patient with documented HSCT failure further excludes delayed transplant-related recovery as a plausible explanation for the observed neurophysiological changes.

A central observation of this study is that the electrophysiological changes were not characterised by simple attenuation or suppression of slow-wave activity, but by a topographical reconfiguration of its generators. In the index patient, independent component analysis and source localisation demonstrated that dominant delta and theta oscillations shifted from fronto-limbic and paralimbic network regions critically involved in autonomic regulation, arousal, and affective processing towards sensorimotor and parietal integrative cortices supporting voluntary action, bodily awareness, and environmental interaction. This redistribution occurred without major changes in overall spectral composition, indicating that pathological oscillatory activity was reallocated within a reorganised network hierarchy rather than eliminated.

This distinction has important implications for understanding functional recovery in severe neurodegenerative encephalopathies. Slow-wave activity is not intrinsically pathological; its clinical significance depends on network dominance and anatomical localisation [11]. When slow oscillations predominate within limbic–frontoinsular systems, they are associated with reduced arousal, impaired consciousness, and autonomic dysregulation. Conversely, reassignment of these oscillatory components to sensorimotor and associative cortical systems implies restoration of top-down cortical modulation over subcortical and limbic activity. The parallel emergence or stabilisation of voluntary behaviour, swallowing, and environmental engagement observed in our patients provides convergent functional support for this network-level interpretation.

The dissociation between structural disease progression and functional recovery observed in this cohort can be interpreted within a bioelectrical framework [12]. While cerebral demyelination defines the anatomical substrate of disconnection, endogenous bioelectrical organisation determines whether residual neural networks operate in a coordinated or collapsed state. Within this framework, structural damage alone is insufficient to fully explain functional outcome at advanced disease stages.

Rather than implying reversal of structural pathology, the present findings are consistent with partial reintegration of surviving circuits through reorganisation of large-scale network dynamics, even in the presence of persistent white matter damage. This interpretation is supported by a growing body of experimental and regenerative biology literature identifying endogenous bioelectric fields as key regulators of cellular behaviour, immune responses, and large-scale tissue organisation in the nervous system. The present clinical and neurophysiological observations extend these principles to human paediatric leukodystrophy.

The convergence of conventional EEG normalisation, quantitative spectral rebalancing, and source-resolved network reallocation across patients occurring both in the absence of haematopoietic stem cell transplantation and following documented HSCT failure argues against spontaneous fluctuation, delayed transplant-related recovery, or vigilance effects as sufficient explanations for the observed changes.

Collectively, the data support a model in which childhood cerebral leukodystrophies involve a progressive breakdown of endogenous bioelectrical coordination that amplifies the effects of metabolic, inflammatory, and structural pathology. Within this framework, targeted restoration of bioelectrical network integrity may mitigate functional collapse, even when primary genetic or structural abnormalities persist, reframing advanced disease not as a static endpoint but as a dynamic state of network-level vulnerability.

This study has limitations inherent to its longitudinal, observational, multi-case design. While numerically limited, the analysis was intentionally designed to maximise mechanistic resolution rather than statistical power, leveraging repeated multimodal neurophysiological assessments to identify conserved patterns of bioelectrical network reorganisation across independent paediatric cases. The small number of patients and absence of an untreated control group reflect both the extreme rarity and the uniformly fatal natural history of advanced childhood cerebral X-linked adrenoleukodystrophy. These methodological constraints are primarily ethical rather than technical. In this clinical context, withholding a potentially biologically active intervention to generate untreated controls is not justifiable; consequently, longitudinal within-subject analysis using objective neurophysiological measures represents the most ethically and scientifically appropriate approach to investigate whether endogenous bioelectrical network disorganisation is reversible in humans [13].

The neurophysiological assessments employed, including conventional EEG, quantitative EEG, independent component analysis, and sLORETA provide direct, reproducible, and mechanistically informative indices of large-scale brain network organisation [14]. The convergence of topographical and spectral changes across independent patients, multiple analytical modalities, and recordings separated by months substantially reduces the likelihood that the observed findings reflect transient state-dependent fluctuations, spontaneous remission, or nonspecific arousal effects.

Within these limitations, the present observations support the feasibility of using multimodal longitudinal neurophysiology as a robust translational framework for probing network-level mechanisms and therapeutic targets in severe paediatric neurodegenerative disorders. The authors’ relationship to the investigated technology is fully disclosed in the dedicated Conflicts of Interest statement.

In conclusion, this longitudinal multi-case analysis indicates that advanced childhood cerebral X-linked adrenoleukodystrophy is not solely a disorder of irreversible structural degeneration but is also characterised by a state of endogenous bioelectrical network disorganisation that remains biologically modifiable. Repeated neuroregenerative treatment was temporally associated with durable clinical stabilisation and reproducible reorganisation of pathological brain rhythms, including a consistent redistribution of slow-wave activity from limbic-dominant networks towards sensorimotor and integrative cortical systems.

Beyond cerebral X-linked adrenoleukodystrophy, converging clinical evidence indicates that REAC RGN-N treatment can also support functional recovery and network-level reorganisation in other childhood leukodystrophies and genetic neurodegenerative disorders, as already documented in paediatric clinical reports [15].

## 5. Conclusions

These observations provide human evidence that large-scale endogenous bioelectrical organisation in paediatric leukodystrophy can undergo directionally adaptive reorganisation, even in the presence of persistent white matter pathology, identifying endogenous bioelectrical coordination as a disease-defining dimension and a biologically grounded therapeutic target in paediatric neurodegeneration.

## Data Availability

All data relevant to the study are included in the article. No additional data are available.
